# Serum Polybrominated Diphenyl Ether (PBDE) Levels Are Higher in Children (2–5 Years of Age) than in Infants and Adults

**DOI:** 10.1289/ehp.0900596

**Published:** 2009-05-06

**Authors:** Leisa-Maree L. Toms, Andreas Sjödin, Fiona Harden, Peter Hobson, Richard Jones, Emily Edenfield, Jochen F. Mueller

**Affiliations:** 1 University of Queensland, National Research Centre for Environmental Toxicology, Coopers Plains, Queensland, Australia; 2 Centers for Disease Control and Prevention, Atlanta, Georgia, USA; 3 School of Life Science, Queensland University of Technology, Gardens Point, Queensland, Australia; 4 Sullivan Nicolaides Pathology, Taringa, Queensland, Australia

**Keywords:** Australia, children, cord blood, human blood serum, PBDEs, polybrominated diphenyl ethers

## Abstract

**Background:**

Polybrominated diphenyl ethers (PBDEs) are used as flame retardants in many products and have been detected in human samples worldwide. Limited data show that concentrations are elevated in young children.

**Objectives:**

We investigated the association between PBDEs and age with an emphasis on young children from Australia in 2006–2007.

**Methods:**

We collected human blood serum samples (*n* = 2,420), which we stratified by age and sex and pooled for analysis of PBDEs.

**Results:**

The sum of BDE-47, -99, -100, and -153 concentrations (∑_4_PBDE) increased from 0–0.5 years (mean ± SD, 14 ± 3.4 ng/g lipid) to peak at 2.6–3 years (51 ± 36 ng/g lipid; *p* < 0.001) and then decreased until 31–45 years (9.9 ± 1.6 ng/g lipid). We observed no further significant decrease among ages 31–45, 45–60 (*p* = 0.964), or > 60 years (*p* = 0.894). The mean ∑_4_PBDE concentration in cord blood (24 ± 14 ng/g lipid) did not differ significantly from that in adult serum at ages 15–30 (*p* = 0.198) or 31–45 years (*p* = 0.140). We found no temporal trend when we compared the present results with Australian PBDE data from 2002–2005. PBDE concentrations were higher in males than in females; however, this difference reached statistical significance only for BDE-153 (*p* = 0.05).

**Conclusions:**

The observed peak concentration at 2.6–3 years of age is later than the period when breast-feeding is typically ceased. This suggests that in addition to the exposure via human milk, young children have higher exposure to these chemicals and/or a lower capacity to eliminate them.

Polybrominated diphenyl ethers (PBDEs) are a class of brominated flame retardants (BFRs) used to reduce the flammability of a multitude of products, including electrical and electronic equipment, plastics, textiles, and furniture. PBDEs are additive chemicals that can leach from products over time and have a tendency to bioaccumulate and bioconcentrate (Martin et al. 2004; McDonald 2002). Their use over the last 30 years has resulted in worldwide contamination of biological and environmental samples (Hites 2004).

Data on adverse health effects due to human exposure to PBDEs are limited. Recent studies, however, have shown PBDE concentrations in human milk to be associated with congenital cryptorchidism (Main et al. 2007) or with lower birth weight, length, and chest circumference (Chao et al. 2007). Thyroid hormone homeostasis in children may be disrupted because of exposure to PBDEs and other contaminants via house dust (Suzuki et al. 2007). Developmental, reproductive, and neurotoxic effects, as well as permanent effects on learning, activity, and behaviors after PBDE exposure have been reported in animals (Birnbaum 2007), with reproductive and developmental toxicity studies showing that fetuses are more sensitive to PBDEs than are adults (Darnerud et al. 2001). Costa and Giordano (2007) state that there is accumulating evidence showing that PBDE exposure in animals causes developmental neurotoxicity at doses similar to those reported in the North American human population.

Data suggest that human exposure to PBDEs occurs in a variety of ways: dietary sources (e.g., Darnerud et al. 2006; Domingo et al. 2008), inhalation in indoor and outdoor environments as well as in cars (e.g., Allen et al. 2007; Mandalakis et al. 2008; Wilford et al. 2005), and dust ingestion (e.g., Sjödin et al. 2008a; Stapleton et al. 2005). Infants and young children receive additional exposure via placental transfer (Bi et al. 2006; Gomara et al. 2007; Mazdai et al. 2003; Schecter et al. 2007), consumption of human milk (Carrizo et al. 2007), and child-specific behaviors, including mouthing of hands and objects possibly contaminated with PBDEs (Stapleton et al. 2008).

PBDEs have been detected in human blood of adults from many countries (e.g., Gomara et al. 2007; Harrad and Porter 2007; Lee et al. 2007; Sandanger et al. 2007; Sjödin et al. 2008b). However, despite the limited scientific evidence suggesting PBDE concentrations in young children are elevated compared with adults (Fischer et al. 2006; Sjödin et al. 2008b; Thomsen et al. 2002), few studies have focused on PBDE concentrations in infants and children. Previously, we observed PBDE concentrations in children 0–4 years of age in Australia to be four to five times higher than concentrations in adults > 16 years of age (Toms et al. 2008), although the use of wide age brackets for pooled samples resulted in an inability to accurately assess the age at which the levels peaked.

The aim of this study was to investigate the age and level of peak PBDE concentrations in the Australian population, with an emphasis on infants and children. Human blood samples were collected from 17 age groups [ranging from newborn (cord blood) to > 60 years] to assess PBDE concentrations. From newborn to 4 years of age, the age brackets covered 6-month periods, followed by 3-year periods for ages 4–15 years and 15-year periods for ages 16 to > 60 years. In addition to samples from children, we collected adult samples in 2006–2007 for temporal comparison with data collected in Australia in 2002–2003 and 2004–2005.

## Materials and Methods

### Samples

We obtained deidentified serum samples from Sullivan Nicolaides Pathology (Taringa, Queensland, Australia) from surplus stored sera that had been collected as part of routine pathology testing. All samples were collected from South East Queensland because previous studies found no geographic differences among pooled samples collected from different regions of Australia (Toms et al. 2008). Before pooling, we stratified all samples according to age and sex. The age groups were as follows: newborn (cord blood), 0–0.5, 0.6–1, 1.1–1.5, 1.6–2, 2.1–2.5, 2.6–3, 3.1–3.5, 3.6–4, 4.1–6, 6.1–9, 9.1–12, 12.1–15, 16–30, 31–45, 46–60, and > 60 years. Age, sex, and date of collection were available for each sample. We calculated the mean age of the pool by taking the mean of all ages of donors included in the pool. This study aimed to investigate PBDE concentration in infants and children, so we used small age ranges for younger age groups and larger age ranges for older age groups where changes in PBDE concentration were previously shown to be less apparent (Toms et al. 2008).

The aim of the sample collection was to combine up to 30 samples per pool with replicate pools per strata where sufficient samples existed [see Supplemental Material, Table 1 (doi:10.1289/ehp.0900596.S1 via http://dx.doi.org)]. Where possible, we used 1 mL of serum from each sample. Because of the small volume of blood obtained from infants and young children, we used < 1 mL in some pools, although we combined the same sample volume from each donor to form a specific pool. We collected a total of 2,420 individual samples and combined them into 84 pools. We used disposable polyurethane pipettes to draw the sample from polypropylene tubes in which the serum was stored. The sample was placed into a 50-mL glass jar and 10 mL was aliquoted into an amber jar for transport to the laboratory.

We used pooled samples because it is the most effective way to obtain large numbers of blood samples from the specific age groups. Difficulties in obtaining parental consent and cooperation of children for blood draws precluded the use of individual samples, as did the volume required for PBDE analysis, which we would have been unlikely to obtain in one blood draw from an infant or young child. Ethics approval was granted on 28 June 2006 by the University of Queensland Medical Research Ethics Committee. The analysis of pooled samples by investigators at the U.S. Centers for Disease Control and Prevention (CDC) was determined not to constitute engagement in human subjects research.

### Analysis

The samples were analyzed at the CDC (Atlanta, GA, USA) with methodology that has been described previously (Sjödin et al. 2004b). Briefly, a set of samples was defined as 24 unknown samples with three analytical blanks and three quality assurance/ quality control samples and processed using a semiautomated sample preparation method. Human sera (2 g) were weighed into test tubes and fortified with internal standards (^13^C-labeled) using a 215 Liquid Handler (Gilson Inc., Middleton, WI, USA). Then formic acid and water were added to denature proteins and dilute the samples on the liquid handler. The target analytes were extracted into dichloromethane using the solid-phase extraction (SPE) workstation (Rapid Trace; Zymark, Hopkinton, MA, USA). Cleanup was performed on a two-layered column. The top layer comprised activated silica and the bottom layer comprised silica gel/sulfuric acid (2:1 by weight). The top layer retained polar lipids such as cholesterol, whereas the bottom layer degraded the remaining lipids to produce an extract free of biogenic material. This procedure was automated using the modular SPE workstation. Samples were evaporated to 1 mL and transferred to the gas chromatograph vials, which were previously spiked with recovery standards. Samples were further evaporated to 10 μL and analyzed by gas chromatography/ high-resolution mass spectrometry with a MAT95XP instrument (ThermoFinnigan, Bremen, Germany). Chromatographic separations were carried out on a 6890 gas chromatograph (Agilent Technologies, Atlanta, GA, USA) fitted with a DB5HT capillary column (15 m, 0.25 mm inner diameter, and 0.10 μm thickness). The following congeners were targeted for analysis: BDE-17, -28, -47, -66, -85, -99, -100, -153, -154, and -183. BDE-209 was not assessed in these samples because the analytical methodology for this congener was not yet fully established. The results are expressed as nanograms per gram of lipid and are reported to two significant figures, using a value of zero for congeners with concentrations below the limit of detection (LOD), which depended on sample size and blanks. ∑_4_PBDE concentrations are the sum of BDE-47, -99, -100, and -153.

### Quality control/quality assurance

To assess the reproducibility of the sampling procedures, we analyzed replicate samples collected in the same strata and compared them. In addition, we included two blind field blanks, which comprised of bovine serum (Sigma Aldrich B8655; Sigma Aldrich, St. Louis, MO, USA) expected to have a PBDE concentration below the LOD with the analytical methodology used. In these blanks, only one congener was present at a concentration slightly above the instrumental LOD, indicating low or negligible contamination during the sample collection and pooling. The congener that was detected in one sample was BDE-99, found at a concentration of 2.9 pg/g serum with an LOD of 2.6 pg/g serum.

### Statistical analysis

Statistical analysis was mostly descriptive, with average concentrations (means or medians) and SDs or ranges reported overall and within age, sex, and collection date groups. We fitted analysis of variance (ANOVA) models (including age group, sex, collection date, and their interaction effects) to test the hypothesis that concentrations differed by overall age group, overall sex, or overall collection date or to test whether age group differences were sex specific. The age groups used in this analysis were as described above, (“Samples”). We used the conventional *p*-value of 5% to report results as statistically significant. We did not include BDE-17, -28, -66, -85, -154, or -183, which were detected in < 60% of samples, in the statistical analysis. We performed statistical analysis using SPSS version 16.0 for Windows (SPSS Inc., Chicago, IL, USA).

## Results and Discussion

We detected PBDEs in all 84 pools of human blood serum; the ∑_4_PBDE concentration ranged from 5.5 to 103 ng/g lipid. Overall, concentrations in children were higher than in adults and varied by age. For adults (both sexes) > 16 years of age, the mean ± SD of ∑_4_PBDEs was 16 ± 18 ng/g lipid. We detected BDE-47, -99, -100, and -153 in all samples, whereas we detected BDE-183, -154, and -85 in 43%, 25%, and 21% of samples, respectively. The concentrations of BDE-47, -99, -100, and -153 across both sexes and all age groups were 2.6–55.1, 0.9–24.2, 0.6–14.1, and 1.3–13.7 ng/g lipid, respectively [for full details, see Supplemental Material, Tables 1 and 2 (doi:10.1289/ehp.0900596.S1)].

We determined the coefficient of variation (CV) using the results within each stratum to compare the reproducibility of sampling procedures between pools. For 70% of strata, the CV between replicates was < 35%. The mean (range) CV was 34% (0.3–103%), 30% (0–100%), 29% (1–106%), and 26% (3–83%) for BDE-47, -99, -100, and -153, respectively. Although variability between replicate pools was evident in some strata, most showed good agreement, indicating that sample collection and pooling procedures were uniform, and it is unlikely that contamination of the samples occurred during sampling. High variability between individuals has been reported previously (Birnbaum and Cohen Hubal 2006; Ryan et al. 2002; Schecter et al. 2003), and variability between replicates is likely attributable to the inclusion in these pools of a few serum samples with an elevated concentration. The distribution of individual results in this study is unknown because only pools have been measured; however, in a U.S. cohort of 2,060 individual serum samples, the reported median concentration of BDE-47 was 19.2 ng/g lipid, with a maximum concentration of 2,350 ng/g lipid (Sjödin et al. 2008b). These levels far exceed the median and would thus have caused the elevated level in a few of the serum pools as observed. Individual variability and a lack of exposure data are limitations of using pooled samples.

### Age

**Figure 1:**
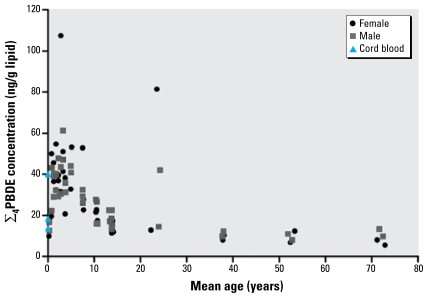
∑_4_PBDE concentrations (ng/g lipid) by mean age (0–80 years) and sex. Each point represents a pool of up to 30 individual blood samples.

For children, mean ∑_4_PBDE concentrations were highest in the 2- to 5-year age groups, with the maximum ∑_4_PBDE concentration (103 ng/g lipid) detected in a pool of serum from females 2.6–3 years of age (Figure 1). We summarized samples into broader age brackets and ran an ANOVA with post hoc Tukey test. The mean ∑_4_PBDE concentrations from cord blood samples and samples from ages 0–2, 6–12, and 13–30 years were similar to each other but were significantly different from concentrations detected in children in the 2- to 5-year age group (Table 1). We observed the lowest concentrations in the adults > 31 years of age, which were also significantly different from those in the 2- to 5-year age group. We detected the lowest ∑_4_PBDE concentration (5.5 ng/g lipid) in a pool from females > 60 years of age. ∑_4_PBDE concentrations in three pools of cord blood ranged from 14 to 40 ng/g lipid, with a mean of 24 ng/g lipid, similar to those in females 15–45 years of age, whose levels ranged from 8.1 to 77 ng/g lipid, with a mean of 27 ng/g lipid. The cord and female blood samples compared here were not fetus–mother pairs, and comparisons should be made with caution because both correlated (Bi et al. 2006; Mazdai et al. 2003) and noncorrelated (Gomara et al. 2007; Meironyté Guvenius et al. 2003) PBDE concentrations in paired cord–maternal blood have been reported.

The results of the present study confirm our previous findings of elevated PBDE concentrations in children 0–4 years of age relative to adults > 16 years of age (Toms et al. 2008). Higher PBDE concentrations in children have been reported in a limited number of studies (Environmental Working Group 2008; Fischer et al. 2006; Thomsen et al. 2002). In contrast, another study found no difference in PBDE concentrations between mothers and their children 7 years of age (Fängström et al. 2005). The present study, however, is the first to use 6-month age brackets for samples ranging in age from newborn (cord blood) to 4-year-olds to determine at what age PBDEs concentrations peak. Increased PBDE concentrations in neonates and infants is most likely attributable to placental transfer and the consumption of human milk (e.g., Noren and Meironyté 2000; Schecter et al. 2003; Toms et al. 2007), with PBDE concentrations in sera of 4-year-olds reportedly higher in breast-fed children than in formula-fed children (Carrizo et al. 2007). In Australia, breast-feeding rates decrease from 85% at birth to 45% at 6 months, 23% at 12 months, and 1% at 24 months (Australian Bureau of Statistics 2003); consequently, for most infants, exposure via this pathway would not typically persist after the first year of life. Despite this pattern, PBDE concentrations appear to increase past 1 year of age. Interestingly, an increasing age trend was not apparent for polychlorinated biphenyls and persistent pesticides in these same samples from birth to 15 years of age (data not shown), indicating that the routes of exposure are different for PBDEs than for traditional persistent organic pollutants. However, we found an increasing trend for polychlorinated biphenyls and persistent pesticides with the subjects’ age for older subjects (data not shown).

We have previously used modeled data, taking into consideration intake and half-lives, to predict the trend of elevated concentrations in Australian infants and young children, but we did not find the magnitude or age of peak PBDE concentrations that we found in the present study (Toms et al. 2008). Reasons for this underestimation may be related to the half-life values used, which may be inaccurate for children, and missing PBDE sources that may contribute to intake levels during early years. The half-lives of PBDEs have been calculated as 1.8, 2.9, 1.6, and 6.5 years for BDE-47, -99, -100, and -153, respectively (Geyer et al. 2004) for non-occupationally exposed adult humans and are based on daily intake and total body burden data. Dust has been suggested as a major source of PBDEs in young children (Jones-Otazo et al. 2005; Lorber 2008; Stapleton et al. 2005), with intake of BDE-47 via dust in Australian 2.5-year-olds estimated at 1–140 ng/day (assuming a daily dust intake rate of 50–100 mg) (Sjödin et al. 2008a). Food intake of tetra-BDEs, of which BDE-47 is the major congener, is estimated at 21 ng/day for this age group (Food Standards Australia and New Zealand 2007). However, variability in dust intake estimates (U.S. Environmental Protection Agency 1997) renders calculation of PBDE intake via this pathway less certain. In addition, BFRs have been detected in baby products such as car seats (Gearhart et al. 2007) and may result in exposures specific to young children. The pathways and level of exposure from such sources require further investigation.

Similar to previous reports (Bradman et al. 2007; Gomara et al. 2007; Harrad and Porter 2007; Julander et al. 2005; Lee et al. 2007), we observed no apparent change in adult PBDE concentration with age.

### Sex

For all age groups combined, the mean ± SD ∑_4_PBDE concentrations in males was 26 ± 13 ng/g lipid and in females was 28 ± 20 ng/g lipid. For adults > 16 years of age, the mean ± SD ∑_4_PBDE concentration for males was 15 ± 11 ng/g lipid and for females was 18 ± 24 ng/g lipid. This sex difference was statistically significant (*p* = 0.05) for BDE-153 only. Higher PBDE concentrations in males than in females have been reported previously (Meneses et al. 1999; Schroter-Kermani et al. 2000; Takasuga et al. 2004; Thomsen et al. 2002) and appears to be attributable to placental transfer and elimination during lactation. In contrast, other studies have reported either no difference or higher concentrations in females (Gomara et al. 2007; Schecter et al. 2005).

### Congener profile

The congener profile in all samples was dominated by BDE-47 (mean, 47%; range of contribution to ∑_4_PBDEs, 31–61%), followed by BDE-153 (20%; 7–47%), BDE-99 (17%; 10–33%), and BDE-100 (13%; 10–18%), whereas the remaining BDE congeners contributed < 10% to total PBDEs. Overall, the profile in these samples was similar to those found in studies of human blood from Australia, New Zealand, North America, and Europe (Harrad and Porter 2007; Schecter et al. 2005; Sjödin et al. 2004a; Thomsen et al. 2002; Toms et al. 2008). In cord blood, the profile was also dominated by BDE-47, followed by BDE-99, -153, and -100, which is consistent with findings by Gomara et al. (2007). However, others have found BDE-153 in higher concentrations than BDE-99 (Bi et al. 2006; Meironyté Guvenius et al. 2003).

**Figure 2:**
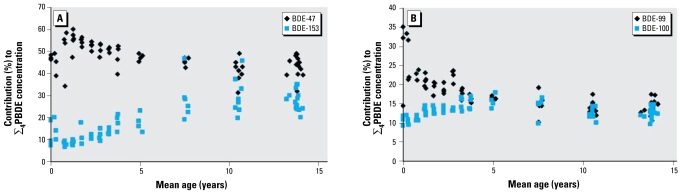
Contribution of BDE-47 and -153 (*A*) and BDE-99 and -100 (*B*) to the ∑_4_PBDE concentration by age (note difference of scale on *y*-axis).

We found no observable differences in profile by sex, except for BDE-153, which made a greater contribution to the ∑_4_PBDE concentration in males than in females. The levels of congener contribution to the ∑_4_PBDE concentration changed with age (Figure 2). The contributions of BDE-47 and -99 decreased with age until around 8 years of age, whereas BDE-100 and -153 increased with age until 5 and 14 years of age, respectively. The half-life of BDE-47 is reportedly shorter than that of BDE-153 (1.8 vs. 6.5 years; Geyer et al. 2004), which may explain the differences in the congener contribution with age. In addition, higher brominated diphenyl ethers are reported to pass the placenta at lower rates compared with lower brominated diphenyl ethers (Bi et al. 2006; Meironyté Guvenius et al. 2003; Schecter et al. 2007), and this may explain why BDE-153 is found in lower concentrations in the younger age groups. Qiu et al. (2009) noted that BDE-99 was more likely to be hydroxylated than are BDE-47 and BDE-100 in humans, which may explain why concentrations of BDE-99 decrease from birth compared with BDE-47 and -100.

### Australian comparison

We compared PBDE concentrations found in this study with those from adult samples collected and pooled in Australia in 2002–2003 and 2004–2005 [Supplemental Material, Figure 1 (doi:10.1289/ehp.0900596.S1)]. Because the age brackets for the younger age groups were different in the two collection periods, we compared samples only from adults > 16 years of age. We found no difference in mean ∑_4_PBDE concentrations for male and female adults > 16 years of age among samples from 2002–2003 (9.9 ± 4.1 ng/g lipid), 2004–2005 (8.1 ± 2.2 ng/g lipid), and 2006–2007 (9.9 ± 2.6 ng/g lipid). Notably, this value for 2006–2007 excluded two elevated results; when we included these, the 2006–2007 mean concentration was 16 ± 19 ng/g lipid. This indicates that despite a ban on the production of penta- and octa-BDE technical products in the European Union in 2004, a phase-out of manufacture of these products in the United States in 2004, and no imports or manufacturing of these pure products in Australia since 2005 (National Industrial Chemicals Notification and Assessment Scheme 2007), the Australian PBDE human body burden has not yet significantly decreased. This is most likely attributable to continued import of products treated with PBDEs, the longevity of products treated with PBDEs (e.g., electrical and electronic equipment, furniture, textiles), and possible exposure after the disposal of these products (Kim et al. 2006). Another factor that may have influenced these findings is the use of different analytical methods for the 2002–2003 and 2004–2005 samples [which were analyzed at Eurofins ERGO GmbH (Hamburg, Germany), as described previously] (Toms et al. 2008), versus the 2006–2007 samples, which were analyzed at the CDC. However, it should be noted that both the laboratories CDC and Eurofins ERGO participate regularly in intercalibration programs in order to certify the analytical measurements [see Supplemental Material for details (doi:10.1289/ehp.0900596.S1)].

### International comparison

International pediatric PBDE data are limited. Where possible, we matched comparisons of data from other countries to age groups in the present study. Because of different reporting practices for sum concentrations, we compared only BDE-47 concentrations. Overall, Australian BDE-47 concentrations in young children were higher than those in European countries but lower than those in the United States.

The mean BDE-47 concentration in Australian children 0–4 years of age was 19 ng/g lipid, which is three times higher than results from a pooled Norwegian sample (*n* = 14; age 0–4 years), in which BDE-47 was detected at 6.2 ng/g lipid (Thomsen et al. 2002). A Menorca Island study found mean BDE-47 concentrations in breast-fed (*n* = 202) and formula-fed 4-year-old subjects (*n* = 42) to be 3.4 and 0.73 ng/g lipid, respectively, whereas the concentration from Australian children was 5–20 times greater, at 16 ng/g lipid (Carrizo et al. 2007). Similar results were found when comparing data from 7-year-old children (*n* = 42) in the Faroe Islands, whose median BDE-47 concentrations were 0.87 ng/g lipid compared with approximately 15 times greater concentration for Australian children at 13 ng/g lipid (Fängström et al. 2005). Individual results obtained in 2004 in California (USA) from one 18-month-old toddler and a 5-year-old girl showed BDE-47 concentrations of 245 and 137 ng/g lipid, respectively (Fischer et al. 2006), whereas data from comparative age brackets in Australia were 21 ng/g lipid for 1–2 year olds and 20 ng/g lipid for 4–6 year olds. Another study from the United States of 20 children ranging in age from 1.5 to 4 years found a median BDE-47 concentration of 30.6 ng/g lipid (Environmental Working Group 2008), which is higher than that found in Australian children of this age (21 ng/g lipid) but lower than those detected in the California samples (Fischer et al. 2006). For older children, the mean BDE-47 concentrations in Australian children 6–11 years of age ranged from 7.0 to 24 ng/g lipid for males and from 5.1 to 14 ng/g lipid for females and were four to eight times lower than those collected from Mexican-American, non-Hispanic black, and non-Hispanic white children 6–11 years of age in 2001–2002 in the United States, whose BDE-47 concentrations ranged from around 60 to 105 ng/g lipid for males and from 65 to 110 ng/g lipid for females (Sjödin A, personal communication). PBDEs have been detected previously in cord blood from other countries. In the present study, the mean ∑_4_PBDE and BDE-47 concentrations in cord blood were 24 ng/g lipid and 12 ng/g lipid, respectively, which is almost 10 times that found in China (Bi et al. 2006) and Sweden (Meironyté Guvenius et al. 2003), around twice that found in Spain (Gomara et al. 2007), but half the concentration reported from Indiana (USA) (Mazdai et al. 2003).

The mean BDE-47 concentrations in Australian adults were higher than those PBDE concentrations in human blood reported from New Zealand, Europe, the United Kingdom, and Asia (e.g., Bi et al. 2006; Harrad and Porter 2007; Schroter-Kermani et al. 2000; Thomas et al. 2006; Thomsen et al. 2002) but lower than those from samples from the United States (Anderson et al. 2008; Sandanger et al. 2007; Schecter et al. 2005; Sjödin et al. 2008b). However, because of different study designs, analytical techniques, and included congeners, any comparisons should be made with caution.

In conclusion, the determination of PBDEs in human blood sera pooled using 6-month age brackets for samples from newborns (cord blood) through to 4-year-olds revealed higher PBDE concentrations in young children compared with infants and adults. To identify the causes of these elevated concentrations, exposure studies should focus on these younger age groups, and an investigation into potential differences in the half-lives of these chemicals in children compared with adults is necessary.

## Figures and Tables

**Table 1:** Mean (95% confidence interval) ∑_4_PBDE (BDE-47, -99, -100, and -153) concentrations (ng/g lipid) by age (years), including outliers, for 2006–2007.

Age (years)	Mean ∑_4_PBDEconcentration (95% CI)	Comparison with 2- to 5-year age group (*p*-value)
Newborn (cord blood)	24 (−11 to 59)*a*	0.047*
0–2	31 (24 to 38)	0.026*
2–6	41 (33 to 49)	—
7–12	26 (20 to 31)	0.002*
13–30	20 (12 to 28)	< 0.001*
> 31	9.4 (7.9 to 11)	< 0.001*

a Three pools only.

* *p* < 0.05.
